# Training on a virtual reality laparoscopic simulator improves performance of live laparoscopic surgery

**DOI:** 10.1111/ases.13005

**Published:** 2021-10-26

**Authors:** Shinji Ohtake, Kazuhide Makiyama, Daisuke Yamashita, Tomoyuki Tatenuma, Masahiro Yao

**Affiliations:** ^1^ Department of Urology Yokohama City University Graduate School of Medicine Yokohama Japan; ^2^ Department of Urology Yokohama Sakae Kyosai Hospital Yokohama Japan

**Keywords:** education, simulator, surgical training

## Abstract

**Introduction:**

To determine whether training laparoscopic nephrectomy (LN) with a virtual reality (VR) simulator improves the performance of porcine LN.

**Methods:**

Twelve urological residents were assigned to two groups: a training and a non‐training group. All participants performed baseline assessments of LN skills and time on the LapPASS® simulator. The training group received preoperative LapPASS® training. Both groups then performed LN using a porcine model. The operations were videotaped and evaluated using the Global Operative Assessment of Laparoscopic Skills (GOALS) system. After porcine LN, the training group performed a final LN with the LapPASS® simulator.

**Results:**

There was no significant difference in the operation time required for porcine LN. There were no significant differences in the total A (autonomy), B (bimanual dexterity), D (depth perception), or T (tissue handling) GOALS scores. However, the total E (efficiency) score in the training group was higher than that in the non‐training group (*P* = .030). The final LN score with LapPASS® was significantly higher than the baseline (*P* = .004).

**Conclusions:**

The results of this study demonstrated that VR LN training improved performance in an actual operation. VR‐based procedural simulation could become a vital part of the laparoscopic training program for residents.

## 1 INTRODUCTION

To ensure patient safety, it is important for surgeons to practice surgical procedures before performing them. In order to improve their skill levels and shorten learning curves and procedure times, surgeons are required to practice procedures outside of the operating room. Simulation tools meet such demands. In the field of urology, laparoscopic surgery is now a standard procedure, but mastering this technique is difficult. Laparoscopic surgery can be practiced using animals, virtual reality (VR) simulators, or with dry boxes. Trainees can experience realistic simulations and bleeding by using animals; however, this approach is expensive, associated with ethical problems, and is not readily available. In dry‐box systems, procedures can be repeated at low cost, but they do not provide a realistic visual field. VR does not suffer from any ethical problems and is easily accessible; however, it does have some disadvantages such as the high initial investment required.

LapPASS®, developed by Mitsubishi Precision, uses VR, and has demonstrated good face and content validity.^1^ The ultimate goal of the surgical training simulator is to improve operating room performance. Various simulators have been developed and evaluated, but few have been evaluated for performance improvement in an actual operating room setting. In this study, we evaluated whether there was a difference in the performance of porcine nephrectomy between a group that was trained with LapPASS® and a group that was not.

## 2 MATERIAL AND METHODS

### 2.1 Study design and participants

This study was approved by the Institutional Review Board of Yokohama City University. The animal experiment was performed in compliance with the Basel Declaration and International Council for Laboratory Animal Science ethical guidelines.

This study was performed between January and June 2019. The subjects were 12 urological residents in their first or second year at Yokohama City University Hospital or other related hospitals. Inclusion criteria were to be a resident and to have not performed any laparoscopic procedure as primary surgeon or simulator training.

**FIGURE 1:**
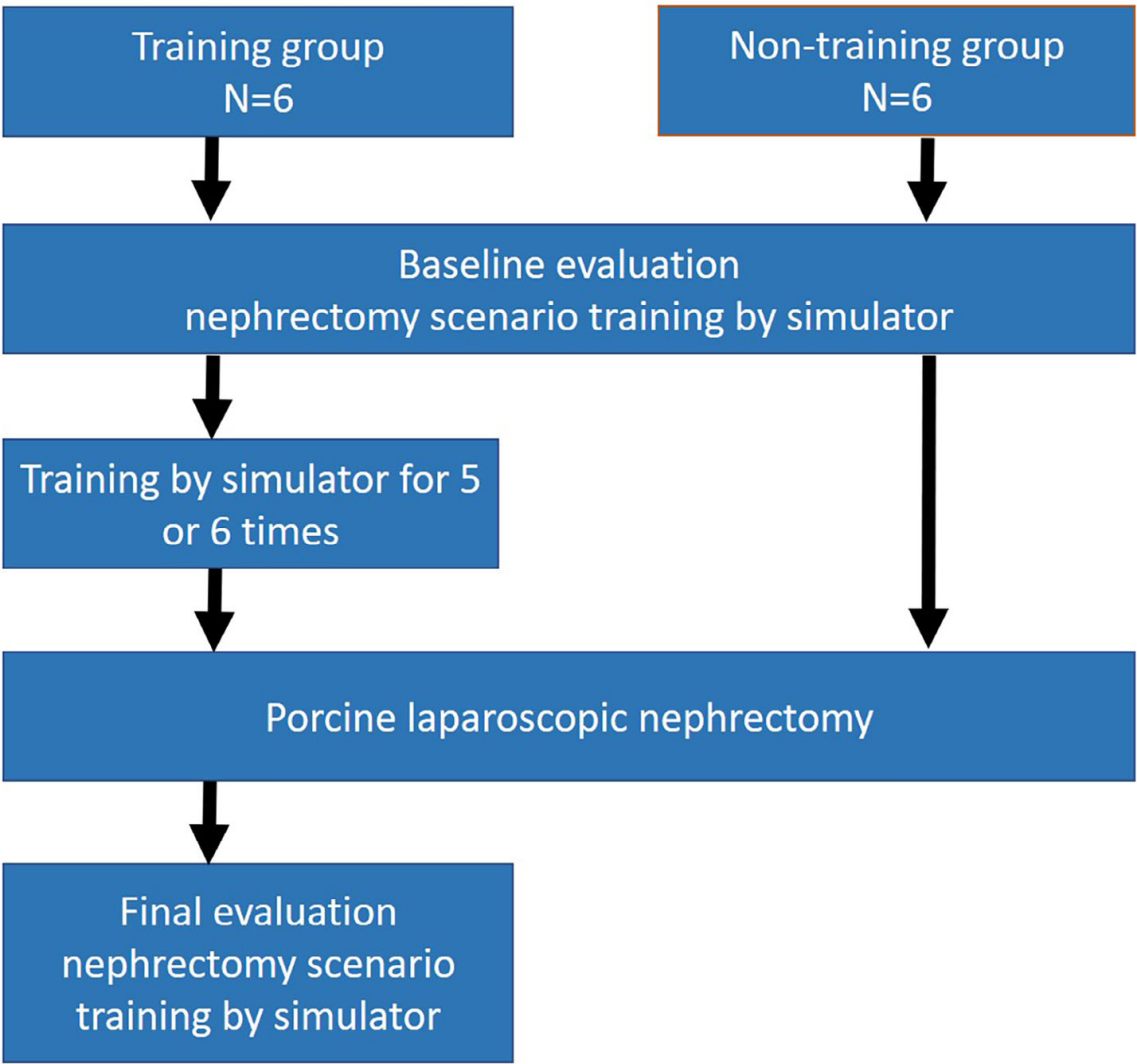
Flow of participants through the trial

Participants signed their informed consent to participate in the study. The data gathered were coded and all reporting was confidential and did not impact the official evaluation. Participants could choose to withdraw at any point during the study and they were made explicitly aware of this at the time of informed consent. They were assigned to the training (n = 6) or non‐training (n = 6) groups. LapPASS® is a VR simulator with a tactile feedback system. The operator can work with two instruments that they can select. Two categories of exercise can be performed on this simulator: four basic exercises (hand‐eye coordination, cutting, clipping, bimanual dexterity) and a complete surgical procedure such as a nephrectomy. Both groups performed baseline assessments of laparoscopic nephrectomy (LN) skills and time on the LapPASS® simulator. Both groups were to continue standard clinical education. During the study no trainee in either group was allowed to perform laparoscopic surgery as a surgeon. The training group attempted all four basic exercises at each session for 4 or 5 hours. All the sessions were supervised by the same teacher whose objective was to have the participants do all the exercises in each program and to intensify their effort for the exercises that they failed. Then, all subjects performed a porcine LN, and their performance was evaluated using the Global Operative Assessment of Laparoscopic Skills (GOALS) system,^2^ a validated assessment tool for laparoscopic surgery, by an experienced surgeon who was blinded regarding the participant groups. After performing porcine LN, the training group was assessed again on the simulator and evaluated. Figure 1 summarizes the flow of participants through the study. The data were coded and all reporting was confidential and did not impact the resident's official evaluation. Participants could choose to withdraw at any point during the study and were made explicitly aware of this at the time of informed consent.

### 2.2 Sample size determination

The power calculation was based on a previous validation study focused on laparoscopic radical nephrectomy.^3^ Participants in the training group were expected to complete a LN in 100 minutes. On the other hand, those in the non‐training group were expected to finish a LN in 140 minutes. The standard deviation of 24 minutes was assumed to be equal in both groups. We set alpha to .05 and power to 0.8, resulting in a sample size of six participants per group.

We determined that with an α of .05 (two‐sided) and a power of 80% (β = .2 giving Zα = 140 and Zβ = 100, largest SD = 24), we required 12 or more trainees.

### 2.3 Data analysis

The primary endpoints were the operating time and GOALS score for porcine LN. As secondary evaluation items, scores on LapPASS® before and after training of the training group were used. Student's *t* test was used for comparison between the groups. All statistical analyses were performed with EZR (Saitama Medical Center, Jichi Medical University, Saitama, Japan),^4^ which is a graphical user interface for R (The R Foundation for Statistical Computing, Vienna, Austria). *P* < .05 was considered significant.

## 3 RESULTS

**FIGURE 2:**
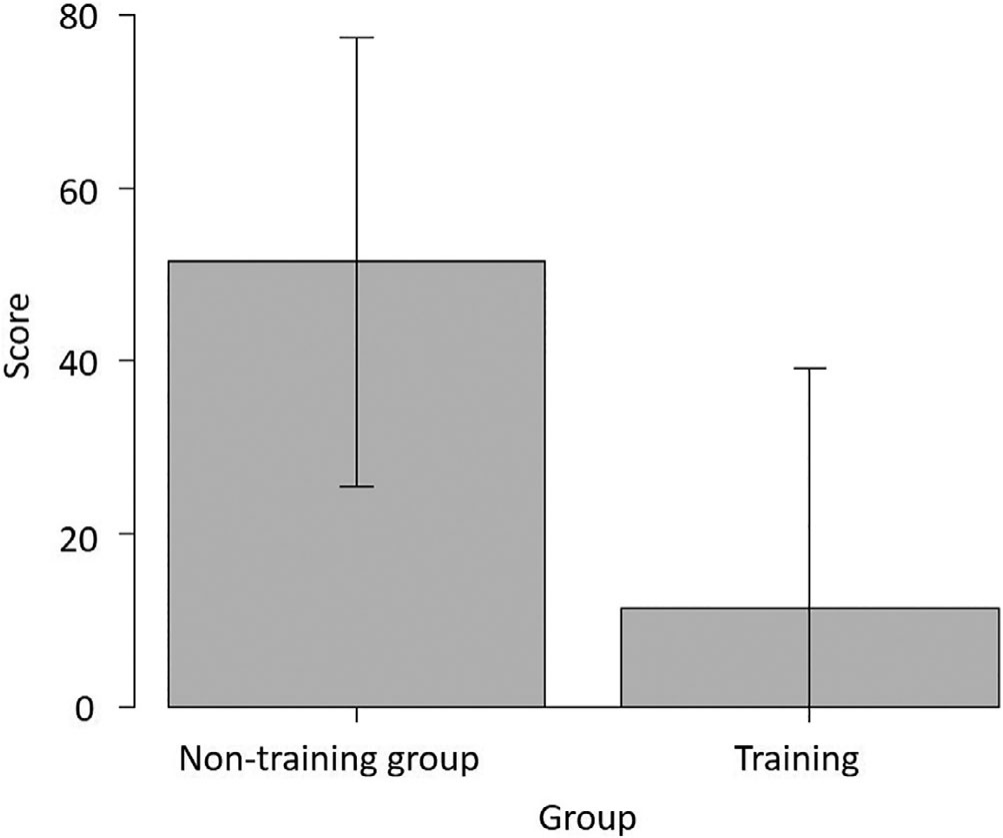
Baseline laparoscopic nephrectomy (LN) score with LapPASS® was higher in the control group than in the training group (*P* = .027)

At the baseline, LN with initial LapPASS® showed a significant difference in surgical scores between the training and non‐training groups (median: 0 [0–68], 59 [0–72], respectively, *P* = .027) (Figure 2). No significant difference was observed for other basic training items (Table 1).

**TABLE 1:** Comparison of simulator‐trained and control groups

		No simulator training	Simulator training	*P*
		(*n* = 6)	(*n* = 6)	
Experience as urologist (y) 1/2/3		3/2/1	3/2/1	1
Gender (male/female)		5/1	4/2	.595
Hand dominance (right/left)		6/0	6/0	1
LN performed as primary		0	0	1
Simulator	Hand‐eye coordination	98 (77–100)	93 (56–99)	.376
	Bimanual dexterity	29 (1–97)	52 (0–88)	.736
	Clipping	70 (28–81)	50 (20–70)	.319
	Cutting	35 (6–49)	31 (4–58)	.907
	Nephrectomy scenario	59 (0–72)	0 (0–68)	.014

*Note*: Data expressed as median. The number of simulator factors investigated was five because of insufficient memory.

Abbreviation: LN, laparoscopic nephrectomy.

**FIGURE 3:**
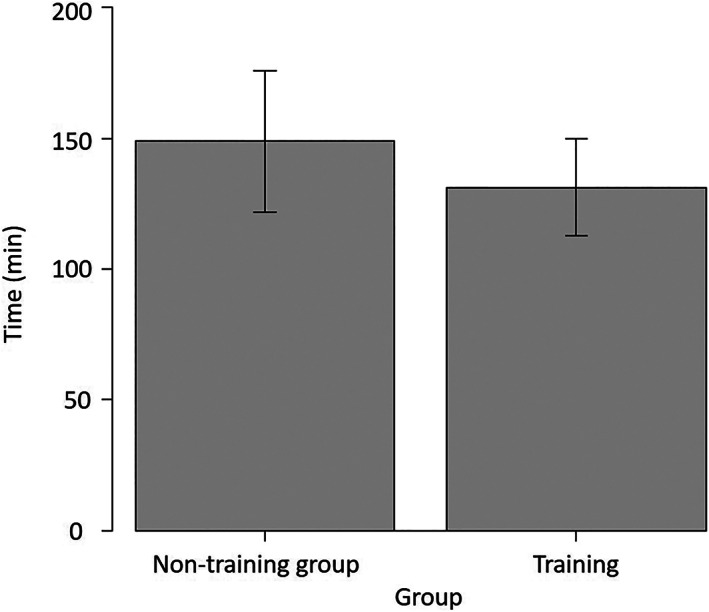
No significant difference in the operation time required for porcine laparoscopic nephrectomy (LN)

**FIGURE 4:**
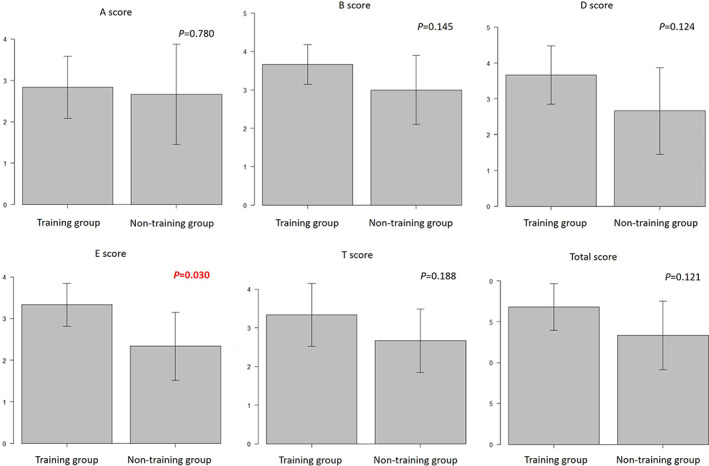
Global Operative Assessment of Laparoscopic Skills (GOALS) scores of porcine laparoscopic nephrectomy (LN). The total E (efficiency) GOALS score in the training group was higher than that in the control group (*P* = .030)

There was no significant difference in the operation time required for porcine LN (Figure 3). There were no significant differences in the total A (autonomy), B (bimanual dexterity), D (depth perception), and T (tissue handling) GOALS scores. However, the total E (efficiency) GOALS score in the training group was higher than that in the non‐training group (*P* = .030) (Figure 4).

**FIGURE 5:**
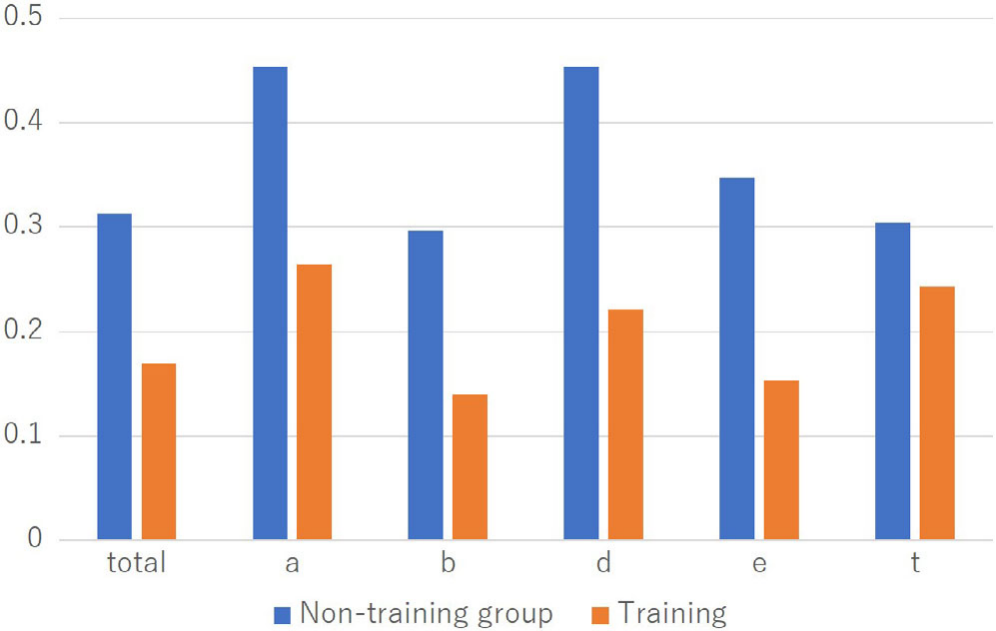
In the training group, all the coefficients of variation (SD/average) of the Global Operative Assessment of Laparoscopic Skills (GOALS) scores were significantly lower than those in the control group (*P* = .005)

In the training group, all the coefficients of variation (SD/average) of GOALS scores were significantly lower than the non‐training group (*P* = .005) (Figure 5) (Table 2).

**TABLE 2:** SD, average, and coefficient of variation of Global Operative Assessment of Laparoscopic Skills (GOALS) score in porcine laparoscopic nephrectomy

	SD		Average		Coefficient of variation		
	Training	Control	Training	Control	Training	Control	*P*
A score	0.7527	1.211	2.833	2.666	0.2647	0.4537	.005
B score	0.5163	0.8944	3.666	3	0.139	0.2966	
D score	0.9164	1.211	3.666	2.666	0.2209	0.4537	
T score	0.8164	0.8164	3.333	2.666	0.243	0.3037	
E score	0.5163	0.8164	3.333	2.333	0.153	0.347	
Total score	2.857	4.1793	16.83	13.33	0.1693	0.3127	

**FIGURE 6:**
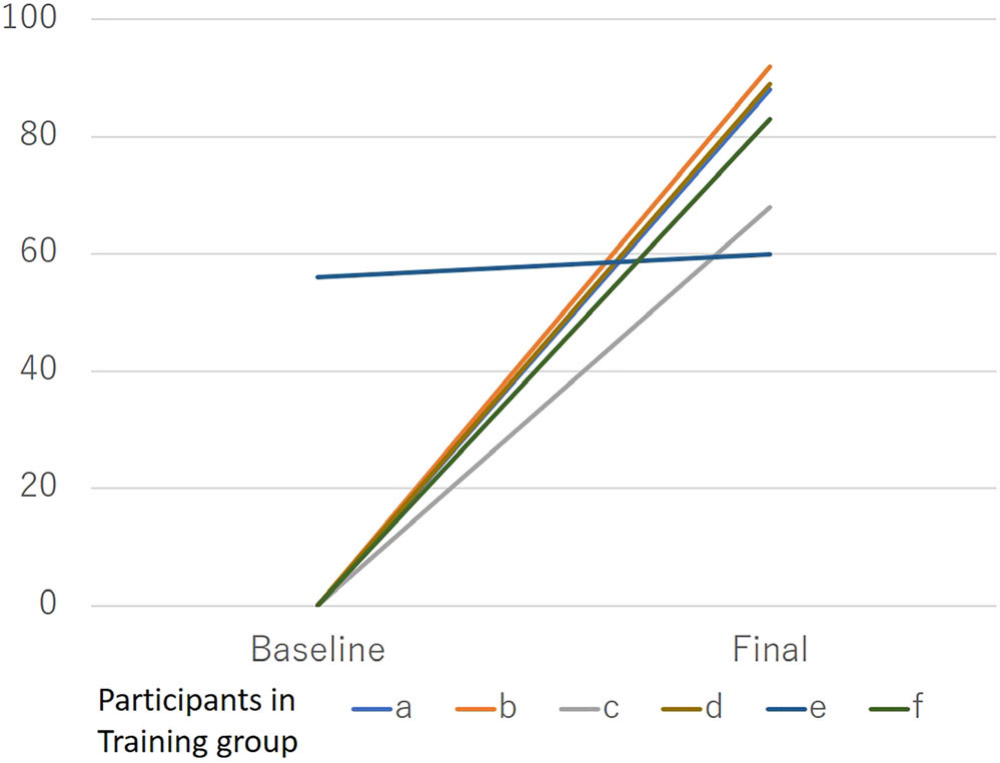
Final laparoscopic nephrectomy (LN) score with LapPASS® was significantly higher than the baseline (*P* = .004)

Comparing the initial and final LapPASS® surgical scores in the training group showed a significant increase of about 70 points (*P* = .004) (Figure 6).

## 4 DISCUSSION

There are various kinds of surgical training methods, and the optimal method should be chosen based on the purpose of the training. There are also several kinds of training tools such as animal models, VR simulators, and box trainers.

Box trainers are superior to other types of simulators in terms of their cost and surgical tools. Box trainers are relatively inexpensive, and such low‐cost options are needed to allow trainees to practice and develop their laparoscopic skills outside of the operating room. In addition, trainees can place various materials in the box trainer and practice a wide range of skills. Suturing is practiced by placing string and cutting by placing paper in the box trainer, and specific procedures, such as peeling, by placing chicken in the box or urethral bladder anastomosis using a balloon model. There are some models to practice specific techniques required in the operation room such as the kidney tumor model made by RICOH that includes a white urinary tract, yellow tumor, and translucent renal parenchyma for the practice of a whole laparoscopic partial nephrectomy.^5^

The major advantage of training with animal models is the realism it provides. Box trainers do not have bleeding or the realistic view of the surgical field that is achieved by training with animal tissue. However, these days there is a tendency to move away from training with animals because of cost, infectious risk, and ethical concerns.

VR simulators include basic skill training software and procedure‐based training software. The main advantage of VR simulators is that every moment of the procedure is recorded. The recorded data can then be analyzed, and trainees' skills can be assessed objectively, which is not possible with box trainers. VR surgical simulations based on patient imaging data represent a form of patient‐specific training. Makiyama et al validated LapPASS® and confirmed that it correctly reproduced anatomical structures, and surgeons felt that it was a useful preoperative training tool.^1^ While expert surgeons do not need to practice standard laparoscopic surgery, patient‐specific simulators allow them to perform patient‐specific preoperative rehearsals, especially for cases with complex anatomy.^6^

Sroka et al reported that training laparoscopic surgery fundamentals in a simulator improved the performance of laparoscopic cholecystectomy.^7^ In other areas, training has been shown to improve actual surgical outcomes. Ahlberg et al reported that a VR training group consistently made significantly fewer errors in laparoscopic cholecystectomy.^8^ Calatayud et al reported that a warm‐up using VR training improved performance in the operation room.^9^ Yang et al evaluated the transferability of laparoscopic skills using a VR simulator (Lap Mentor® https://simbionix.com/simulators/lap-mentor/) and concluded that the training group needed significantly less movement as well as shorter path length.^10^ Larsen et al reported that a VR training (LapSim® https://surgicalscience.com/systems/lapsim/) group performed laparoscopic salpingectomy faster than a control group.^11^ In urology, Lucas et al evaluated whether training on a VR laparoscopic cholecystectomy simulator (Lap Mentor®) improved the performance of LN. They concluded that total Objective Structured Assessment of Technical Skills scores for live porcine LN after training were significantly higher in the training group;^12^ however, the content of the VR training was cholecystectomy, not nephrectomy. Except for Lap Mentor®, there are a few other VR simulators, such as LapSim®, LapVision Smart® (https://www.medvisiongroup.com/lapvision.html) MIST Nephrectomy® (https://www.mentice.com/), and Simendo® (https://www.simendo.eu/), with which trainers can practice simulated LN. Brewin et al evaluated the first VR LN simulator, MIST Nephrectomy®, for its face, content, and construct validity.^13^ Miyata et al validated LapVision Smart® and concluded that it demonstrated good construct validity.^14^ There are so far no reports that nephrectomy scenario training improves performance in the operating room. Although our study used a porcine model, to the best of our knowledge this is the first study to determine whether there is a difference in live LN with or without VR nephrectomy training.

The training group had a significantly lower coefficient of variation of the GOALS score. The coefficient of variation indicates the variation of the data relative to the average value. In the training group, all the coefficients of variation were lower than those in the non‐training group. Furthermore, all the subjects in the training group improved their skills. This indicates the usefulness of this simulator as a training tool.

Although the initial LapPASS® score of the training group was lower than that of the non‐training group, after training, the training group overcame the difference in total GOALS score. Furthermore, they achieved a higher E score in GOALS than the non‐training group. Thus, skill in laparoscopic surgery and performance in an actual operation were improved by VR simulator training. Based on these results, we recommend that urological residents train with LapPASS. This approach would decrease training cost, increase training opportunities, and improve patient safety.

This study has some limitations. First, the subjects were not randomized. The training group included residents at Yokohama City University Hospital, and the non‐training group consisted of residents from a related hospital that could not frequently perform the training. During LN training in LapPASS®, if there was excessive bleeding, the scenario was stopped at that timing and the score became 0. In the training group, five out of six subjects injured the vasculature during the scenario and scored 0 points. On the other hand, in the non‐training group, five out of six subjects completed the scenario. We divided all participants into two non‐random groups before the first VR simulation, and this may explain why there was a statistical difference in the first evaluation. The participants performed on and off training; however, we think its effect on this study was small because this was a short‐term study and all of them had not performed any laparoscopic procedure or simulator training.

Second, the observation period was short. Surgical techniques require long‐term repeated practice. In this trial, the period was limited, so it was not possible to evaluate the improvement of performance and the learning curve. Furthermore, there are various urological operations other than nephrectomy. The techniques used are extensive, and it is difficult to evaluate the entire surgical procedure by nephrectomy alone. A long‐term evaluation with a larger number of subjects and various procedures is necessary.

## CONFLICT OF INTEREST

The authors declare they have no conflicts of interest to declare and received no financial support for this study.

## AUTHOR CONTRIBUTION

Designed the study: Shinji Ohtake, Kazuhide Makiyama, Daisuke Yamashita, Tomoyuki Tatenuma, and Masahiro Yao. Collected data: Shinji Ohtake. Managed the animal experiment: Shinji Ohtake and Kazuhide Makiyama. Performed the statistical analysis: Shinji Ohtake. Wrote the manuscript: Shinji Ohtake and Kazuhide Makiyama. All authors read and approved the final manuscript.

## ETHICS STATEMENT

This study was approved by the Institutional Review Board of Yokohama City University. The animal experiment was performed in compliance with the Basel Declaration and International Council for Laboratory Animal Science ethical guidelines.

## Data Availability

The data that support the findings of this study are available from the corresponding author upon reasonable request.
